# From competencies to action: unpacking the path from students' sustainability competencies to self-reported prosocial behavior

**DOI:** 10.3389/fpsyg.2026.1918048

**Published:** 2026-09-03

**Authors:** Katarzyna Piwowar-Sulej, Katarzyna Tracz-Krupa, Vincent Cassar, Frank Bezzina

**Affiliations:** 1 Faculty of Business and Management, Wroclaw University of Economics and Business, Wroclaw, Poland; 2 Faculty of Business and Management, Wroclaw University of Economics and Business, Wroclaw, Poland; 3 Department of Business and Enterprise Management, University of Malta, Msida, Malta; 4 Department of Business and Enterprise Management, University of Malta, Msida, Malta

**Keywords:** change agents, competencies, higher education, sustainability, sustainable development goals

## Abstract

**Introduction:**

Achieving social sustainability requires a deeper understanding of how individual competencies are associated with active prosocial actions. This cross-sectional study examined the associations between higher education students' key competencies in sustainability (KCiS) and their self-reported prosocial behavior, focusing on the mediating role of prosocial norms, and the moderating influence of individual cultural orientations (horizontal and vertical collectivism).

**Methods:**

Grounded in Norm-Activation Theory and informed by Social Learning Theory, structural equation modeling (SEM) and bootstrap moderated mediation analysis were conducted on survey data from 883 university students across five European universities.

**Results:**

Results indicated that prosocial norms indirectly connected KCiS with prosocial behavior. Contrary to expectations, horizontal collectivism did not moderate the direct or indirect pathways. However, a significant negative interaction emerged: vertical collectivism attenuated the positive association between KCiS and prosocial norms, with the conditional indirect association weakening at higher levels of vertical collectivism.

**Discussion:**

Testing conditional indirect associations revealed a dampening pattern that standard bivariate models overlook. Contextualized by self-reported data, these findings demonstrate how sustainability competencies and cultural variations relate to prosocial action, offering theoretical insights into cultural boundary conditions and informing targeted higher education strategies.

## 1 Introduction

In the face of unprecedented global challenges, ranging from social inequalities to environmental crises, sustainable development has become increasingly urgent. These challenges threaten ecosystems, food security, public health, and socio-economic stability, disproportionately affecting marginalized communities. The growing focus on sustainability reflects a broader understanding that economic, environmental, and social issues are interconnected (Barron et al., 2025). However, social sustainability-often linked to the UN's Agenda 2030 and its 17 Sustainable Development Goals (SDGs)-remains a relatively new concept in policy and academic discussions (Nilsson Dahlström, 2025; Wang et al., 2024). Unlike the environmental and economic dimensions, it has received less attention; yet it is equally crucial. Social sustainability emphasizes justice, inclusion, equity, democracy, social capital, and human skills, aiming to create cohesive societies where all individuals can thrive (Barron et al., 2025). In the context of higher education, it underscores the role of universities in promoting diversity, equity, and student wellbeing, preparing students not only for the workforce but also to engage responsibly with their communities (Barrio Campo et al., 2026; Nilsson Dahlström, 2025; Rey-Garcia and Mato-Santiso, 2020). The first step for students to become change agents is that they should demonstrate prosocial behavior, defined as voluntary actions intended to benefit others or society, including helping those in need, engaging in social activities, and making ethical choices (Kim and Kim, 2022). Therefore, there is a need to identify factors that are systematically associated with such behaviors.

Past research determined that emotional wellbeing (Hidayah et al., 2026), childhood experiences (Zhou et al., 2024), mortality salience (Chang et al., 2025), technostress and thankfulness (Abyadh, 2025), and combinations of academic learning with real-world activities (Nikolskiy et al., 2025) are strongly associated with prosocial behavior among university students. In turn, the general literature highlights that competencies are associated with individual behavior (Furlotti et al., 2022). Although key competencies in sustainability (KCiS) are understood as integrated sets of knowledge, skills, and attitudes that support effective performance and problem-solving in relation to real-world sustainability challenges and opportunities (Wiek et al., 2011), there is still a gap in the literature regarding how, and under what conditions, they relate to students' prosocial behavior. This study aims to fill this gap by examining prosocial norms as a mediating mechanism and individual-level collectivism as a contextual variable in the association between KCiS (Bezzina et al., 2025) and students' self-reported prosocial behavior. To underpin the relationships between variables, the authors applied Social Learning Theory (SLT) and Norm-Activation Theory (NAT) as an explanatory framework.

At this point it is worth describing further gaps that will be addressed by this study. As Kim and Kim (2022) demonstrated, a large body of studies focus on the link between social norms (norms functioning in a society) and individual behavior, leaving the space for examining individual prosocial norms. Drawing on NAT (Schwartz, 1977), individual prosocial norms, defined as internalized moral obligations to act in ways that benefit others (Siu et al., 2012), are positively associated with action when linked with individual capabilities such as KCiS. In addition, authors studying the formation of prosocial norms emphasize that, according to SLT, education plays a crucial role in aligning these norms among young people (Siu et al., 2012). Thus, this study aims to verify if KCiS are positively associated with individual prosocial norms, and indirectly with students' prosocial behavior.

Another important aspect, emphasized in the literature (Piwowar-Sulej, 2022), is the need for more research on how cultural characteristics are related to sustainability-related responses of individuals and organizations. Cultural characteristics, particularly collectivist orientations prioritizing group wellbeing over individual goals (Hofstede, 1984), strongly correspond to individuals' concern for others. Individualism focuses on personal rights and individual goals, viewing people as autonomous actors with their own preferences, abilities, and ideas. In contrast, collectivism prioritizes the wellbeing of the group as a whole (Hofstede, 1984). Although researchers agree that in collectivism-oriented cultures each member has specific duties that contribute to the mechanism of interrelated groups and the larger holistic society more than in those which are more individualism-oriented (Cheng et al., 2020), Khalil et al. (2021) contend that research examining the role of culture at the macro (national) level faces challenges in terms of external validity, because culturally responsive and context-sensitive perspectives within a country may vary. Therefore, this study further aims to explore whether the individual's sense of collectivism acts as a moderator of the association between KCiS and prosocial norms as well as conditions the entire indirect association from KCiS to prosocial behavior.

Considering the above, this study makes several contributions to the literature. First, it addresses key conceptual gaps in prior empirical models. While Kim and Kim (2022) demonstrated the importance of distinguishing societal norms from individual moral obligations, adjacent empirical research often focuses on domain-specific constructs like environmental identity (limited to ecological actions), general moral obligations, or Public Service Motivation. In contrast, prosocial norms capture generalized, internalized socio-moral obligations applicable across diverse interpersonal and community settings. Prosocial norms are uniquely suited here because KCiS equip students to evaluate systemic interdependencies, which directly corresponds to personal responsibility across broad social domains. Second, this study positions NAT as its main tested framework-viewing KCiS as a framework factor corresponding to personal norms–while using Social Learning Theory as a complementary interpretive lens to contextualize competency acquisition, responding to calls for multi-theoretical approaches (Iqbal and Piwowar-Sulej, 2023). Third, it addresses cultural considerations by exploring how individual-level collectivism moderates the association between KCiS and prosocial norms, moving beyond limitations of national-level cultural research (Khalil et al., 2021). To capture disaggregated cultural dynamics, this study separates collectivism into horizontal collectivism (prioritizing equality and mutual interdependence) and vertical collectivism (prioritizing hierarchy, duty, and deference to authority), examining their distinct moderating pathway from KCiS to prosocial norms. Finally, most studies on the development of sustainability-related competencies and behaviors have been conducted within a single country or even a single university (e.g., Schrage et al., 2025; Woo et al., 2025). In contrast, this study draws on a diverse sample of 883 students from five universities across multiple countries (Poland, Kosovo, Czech Republic, Albania, and Spain), providing exploratory cross-cultural breadth across distinct European higher education contexts, rather than asserting universal generalizability.

Finally, because all variables in this study were assessed via self-report measures, the findings reflect participants' self-assessed capabilities, espoused cultural values, and reported behavioral tendencies rather than objective skill assessments or direct behavioral observations.

## 2 Theoretical background and hypotheses

This study draws on NAT and SLT to conceptualize how KCiS are associated with prosocial behavior. NAT posits that prosocial behavior is systematically aligned with active personal norms, which correspond to instances where individuals are aware of the consequences of their actions and feel responsible for addressing them (Schwartz, 1977; Stern, 2000). In parallel, SLT suggests that such norms and behaviors are learned and reinforced through social and educational processes, including education, observation, and experience (Bandura, 1977; Siu et al., 2012). Together, these frameworks suggest that sustainable competencies developed through education are theoretically linked with internalized norms, which co-occur with prosocial behavior.

### 2.1 The relationship between KCiS and prosocial norms

KCiS encompass systems thinking, anticipatory, normative, strategic, and interpersonal competencies (Wiek et al., 2011). These competencies equip individuals with the ability to understand complex societal challenges, anticipate consequences, and evaluate actions based on ethical considerations. According to NAT, personal norms are theorized to be expressed in the presence of awareness of consequences-the cognitive recognition that one's actions or inactions can produce harm or benefit to others-and ascription to responsibility-the feeling of personal agency or responsibility for those outcomes (Schwartz, 1977; Stern, 2000). While awareness of consequences and ascription to responsibility are omitted as direct mechanisms in this study, KCiS is positioned as a conceptual framework factor. Conceptually, systems thinking and anticipatory competencies align with cognitive foresight (aligning with awareness of consequences), while normative, strategic, and interpersonal competencies co-occur with personal agency and duty (reflecting ascription to responsibility).

Complementarily, SLT suggests that educational experiences correspond to the internalization of values and norms through cognitive and social processes (Siu et al., 2012). Empirical research indicates that competencies and knowledge structures are systematically aligned with attitudes and normative beliefs that guide behavior (Furlotti et al., 2022; van der Linden et al., 2015). Thus, KCiS should correspond to higher levels of individuals' prosocial norms. Therefore, we posit that:

*H1: KCiS are positively associated with prosocial norms*.

### 2.2 The relationship between prosocial norms and prosocial behavior

Prosocial norms represent internalized moral obligations that are systematically aligned with behaviors benefiting others (Siu et al., 2012). According to NAT, when active, these norms are directly associated with behavior by reflecting a sense of personal responsibility to act (Schwartz, 1977). This perspective is supported by extensive empirical research demonstrating that personal norms are strong predictors of prosocial and environmentally responsible behavior (Kim and Kim, 2022; Stern, 2000).

Importantly, such moral obligations are not reported in isolation. From the SLT perspective, individuals align with prosocial norms through observational learning, modeling, and reinforcement processes (Bandura, 1977, 1986). By observing significant others (e.g., peers, leaders, or societal role models) engage in helping and cooperative behaviors that are socially rewarded, individuals internalize these behaviors as appropriate standards of conduct. Over time, these socially acquired standards reflect internalized moral obligations that co-occur with behavior even in the absence of external reinforcement.

When prosocial norms are active, individuals perceive helping others or contributing to societal wellbeing as a moral obligation, which corresponds to higher reporting of behaviors such as volunteering, cooperation, and ethical decision-making (Hoffman, 2000; Schwartz, 1977). Thus, prosocial norms function as a proximal statistical correlate of prosocial behavior, operating through both internalized moral responsibility and socially-learned behavioral standards. Therefore, we hypothesize that:

*H2: Prosocial norms are positively associated with prosocial behavior*.

### 2.3 The indirect association of prosocial norms between KCiS and prosocial behavior

Integrating the above arguments, KCiS are expected to correspond to behavior indirectly through prosocial norms. NAT explicitly posits that competencies and awareness are associated with behavior primarily through personal norms (Schwartz, 1977; Stern, 2000). In this sense, prosocial norms function as a proximal factor connecting competencies with behavior.

From a complementary SLT perspective, individuals also align sustainability-related competencies and prosocial norms through observational learning, modeling, and reinforcement processes (Bandura, 1986). Exposure to role models, organizational practices, and socially rewarded sustainable behaviors corresponds to the internalization of normative expectations, thereby aligning both competency development and the presence of prosocial norms. Over time, these socially learned standards reflect internalized moral obligations that are systematically aligned with behavior.

Individuals with higher KCiS are more likely to exhibit stronger prosocial norms, which co-occur with higher levels of prosocial actions. Prior research supports this indirect pathway, indicating that cognitive and competency-based factors are systematically aligned with behavior alongside normative and attitudinal factors (van der Linden et al., 2015; Morrison, 2011). Together, NAT and SLT suggest that prosocial norms serve as an indirect bridge connecting KCiS to behavior. Therefore, we postulate that:

*H3: Prosocial norms mediate the association between KCiS and prosocial behavior*.

### 2.4 Moderating role of individual-level collectivism

Cultural values condition how individuals interpret and align KCiS with prosocial norms (Hofstede, 1984; Triandis, 2001). Specifically, individuals high in collectivism tend to define the self in relational terms and exhibit greater sensitivity to group obligations, social expectations, and normative alignment with in-groups (Oyserman et al., 2002).

From a theoretical standpoint, individual-level collectivism does not merely correspond to whether prosocial norms are reported, but rather conditions the degree of association between KCiS and norm expression. Specifically, individuals with stronger collectivist orientations are more likely to interpret their competencies through a social-relational lens, whereby knowledge and skills are framed as resources that should be used for collective benefit. In contrast, individuals with lower collectivist orientations may view competencies more individually, attenuating the alignment between competencies and moral or socially oriented obligations.

Accordingly, vertical and horizontal collectivism are expected to condition the positive association of KCiS with prosocial norms, such that the association is stronger among individuals with higher levels of collectivism. This aligns with evidence showing that collectivist values correspond to heightened sensitivity to social norms, higher norm-consistent behavior, and greater concern for collective welfare (Cheng et al., 2020; Kim and Kim, 2022; Markus and Kitayama, 1991; Triandis, 2001). Because horizontal collectivism emphasizes peer equality and shared goals while vertical collectivism stresses social hierarchy and duty, these dimensions may reflect distinct psychological alignments with norm expression by connecting individual competencies with group-oriented motives and social accountability.

While a collectivist orientation could plausibly condition the norm-to-behavior path, we position collectivism primarily as a moderator of the first-stage relationship between KCiS and prosocial norms. Grounded in SLT and NAT, cultural orientations serve as fundamental interpretive lenses through which individuals process and internalize acquired competencies. Collectivism frameworking reflects how individuals evaluate their personal capabilities in relation to social responsibility; specifically, collectivist values condition whether knowledge and systemic thinking (KCiS) correspond to collective obligations rather than purely individual skill sets. Once a personal norm is internalized and activated, it operates as a potent, immediate self-regulatory factor that is directly associated with behavior relatively independent of cultural self-construal. Therefore, collectivism is conceptualized as playing its primary moderating role during the formative stage-modulating how competencies correspond to internalized moral norms-rather than during the downstream behavioral phase. Therefore, we postulate that:

*H4a: Individual-level horizontal collectivism moderates the relationship between KCiS and prosocial norms, such that the positive association is stronger at higher levels of horizontal collectivism*.

*H4b: Individual-level vertical collectivism moderates the relationship between KCiS and prosocial norms, such that the positive association is stronger at higher levels of vertical collectivism*.

### 2.5 Moderated mediation

Building on the moderating interaction of collectivism on the association between KCiS and prosocial norms (H4a), collectivism is further expected to condition the indirect association between KCiS and prosocial behavior through prosocial norms. Specifically, because prosocial norms function as the proximal motivational factor aligned with competencies in NAT (Schwartz, 1977; Stern, 2000), factors associated with higher norm expression will also correspond to a stronger overall indirect association.

From an SLT perspective, individuals align with norm-guided behavior through social modeling and the internalization of observed expectations (Bandura, 1986, 1991). In collectivist contexts, where social referents and in-group expectations are more salient, socially learned standards are more strongly integrated into self-regulatory systems, thereby strengthening the alignment between competencies, normative obligations and subsequent behavior.

Accordingly, because H4 indicates that collectivism conditions the strength of the association between KCiS and prosocial norms, this moderating interaction extends to the indirect pathway. In line with conditional process theory, when a moderator conditions the first stage of a mediation model, the magnitude of the indirect association is systematically conditioned by that moderator (Hayes, 2017). Thus, the indirect association between KCiS and prosocial behavior via prosocial norms is expected to vary across levels of collectivism. Therefore, we hypothesize that:

*H5a: Individual-level horizontal collectivism moderates the indirect association between KCiS and prosocial behavior via prosocial norms, such that the indirect association is stronger at higher levels of horizontal collectivism*

*H5b: Individual-level vertical collectivism moderates the indirect association between KCiS and prosocial behavior via prosocial norms, such that the indirect association is stronger at higher levels of vertical collectivism*

**Figure 1:**
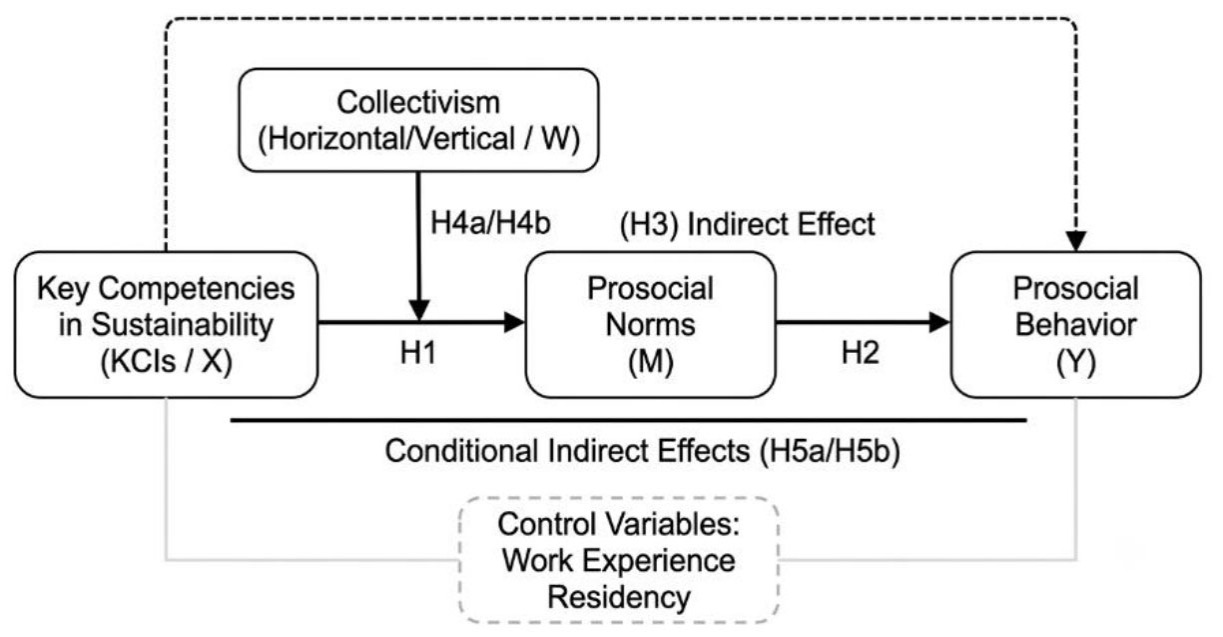
Hypothesized moderated mediation model.

The Hypothesized Moderated Mediation Model adopted in this study is illustrated in Figure 1.

Given prior evidence that socio-environmental contexts are systematically associated with prosocial norms, behavioral tendencies, and sustainability-related attitudes (Gelfand et al., 2011), residency (0 = village/rural, 1 = town/city) was selected a priori as a demographic control in both models. Work experience (0 = no prior experience, 1 = prior experience) was likewise included prior to the analysis as a covariate, as accumulated professional exposure corresponds to differences in normative orientations and behavioral tendencies through processes of socialization and organizational learning (Bardi and Schwartz, 2003).

## 3 Material and methods

### 3.1 Sampling

This study focused on business and economics students enrolled across five universities located in Poland, the Czech Republic, Albania, Spain, and Kosovo. These institutions were participating in an international student mobility and collaboration project funded by the Polish National Agency for Academic Exchange (NAWA) at the time of study. The inclusion of multiple universities from different national contexts was intended to capture broader contextual diversity across higher education settings, rather than to conduct institutional comparisons or produce findings representative of all students within each higher education institution or country.

Ethical approval and data collection clearance across all five participating universities were granted under the lead university's ethics committee (Protocol code: EZD: RN-CBN-SDB.0021.06.2026; see ethics statement for details). Permission to conduct the study was also secured from the respective deans of the participating institutions. Prior to accessing the survey, students viewed an online information page detailing the study's purpose, risks, and voluntary nature. Participants actively indicated their informed consent by selecting an “I Agree to Participate” button before the questionnaire was rendered. Subsequently, faculty coordinators distributed an invitation email containing a link to an online questionnaire to all registered undergraduate and postgraduate students.

This study targeted all undergraduate and master's students within the participating institutions who were able to understand English, as the questionnaire was administered in English. This approach aimed to include the entire accessible population of eligible students while respecting participants' autonomy and informed consent (Bryman, 2016). However, voluntary participation may introduce self-selection bias, and limiting participation to English-proficient students may have excluded some individuals, potentially affecting generalizability. Participant anonymity and confidentiality were assured throughout the study.

Determining appropriate sample sizes for moderated mediation models is inherently complex, as requirements depend on effect sizes and model specification, rather than fixed rules of thumb (Xu et al., 2024). Monte Carlo simulation-based research demonstrates that detecting indirect and interaction effects typically requires larger samples than simple mediation models, with approximately 100–200 observations sufficient for large effects, 200–300 for medium effects, and 400–800 or more for small effects (Schoemann et al., 2017).

University coordinators were asked to obtain a minimum of 150 responses per institution. Data collection occurred during May 2026 through an online platform that mandated full completion. To account for shared campus-Wi-Fi networks, duplicates were only flagged when matching IP addresses were accompanied by identical submission timestamps and identical response vectors. Careless responses (completion times < 3 minutes) were also removed, yielding a final clean sample of 883. This sample provided sufficient statistical power to detect even relatively small effects (Schoemann et al., 2017). Because exact population counts were not provided by participating institutions, response rates could not be calculated. The sample was distributed across five public education institutions located in Poland (19.4%), Kosovo (20.3%), the Czech Republic (20.2%), Spain (19.4%), and Albania (20.7%). The sample consisted predominantly of female respondents (56.4%). Most participants were enrolled in undergraduate programs (76.7%), with the remainder pursuing master's degrees (23.3%). A large majority of the respondents reported that they had not undertaken internships (75.7%) or formal employment (58.9%). The mean age of participants was 22.1 years (SD = 4.8), with ages ranging from 18 to 57. Comparisons between the sample and the broader student population were not possible due to the unavailability of institutional demographic data.

It is also important to note that at the time of study, the participants were not enrolled in programs explicitly designed to develop KCiS. Consequently, their responses are likely to reflect prior awareness or informal exposure to sustainability concepts rather than outcomes of structured educational interventions.

### 3.2 Measures

Data were collected via a self-administered online questionnaire comprising two sections. The first section gathered demographic information, including gender, age, university affiliation, and level of study. Participants were also asked about their internship and work experience, as well as their awareness and engagement with sustainability. Specifically, items assessed whether respondents understood sustainable development, their level of knowledge in sustainability, whether they possessed and applied sustainability-related competencies (e.g., strategic or systems thinking) in the labor market, their interest in attending sustainability competency workshops, and whether their university or faculty offers specialized courses in sustainable development. The second section included 39 Likert-type items ranging from 1 (strongly disagree) to 5 (strongly agree) using validated scales.

KCiS were measured using the 15-item KCiS scale developed by Bezzina et al. (2025). A sample item is: “I can construct action plans that solve sustainability problems.” Cronbach's α is 0.92.

The prosocial norms scale was adapted from Bezzina et al. (2025) by broadening its focus from environmental protection to general societal welfare, grounded in the Norm Activation Model (Schwartz, 1977). The three items evaluate personal obligation (“I feel a personal obligation to support people in my community who are in need”), normative corporate expectations (“I believe that businesses should actively contribute to solving societal problems”), and daily behavioral consideration (“I feel obliged to consider societal needs in my daily behavior”). Following consultation with an expert in the field, the second item was specifically adapted from its original framing (“government pressure on industry”) to corporate responsibility (business involvement). This adaptation isolates direct expectations of corporate social responsibility within a business and economics student cohort, preventing political attitudes toward state regulation or government policy from confounding the measure. Conceptually, this item reflects an extension of an individual's personal moral orientation to institutional agents; in populations of business and economics students, personal moral obligations toward societal welfare naturally encompass normative expectations regarding corporate civic duty. Empirically, a post-hoc item-total reliability analysis confirmed exceptional scale cohesion, with the corporate expectations item demonstrating the highest corrected item-total correlation (*r* = 0.70, exceeding the 0.50 threshold) and the largest contribution to overall internal consistency (Cronbach's α is 0.83, dropping to 0.74 if deleted).

The prosocial behavior scale was taken from the four-item traditional prosocial behaviors scale by Kim and Kim (2022). A sample item is: “I help people other than students who are worse off than me.” Cronbach's α is 0.80.

Collectivism was assessed using eight items from Triandis and Gelfand (1998), comprising two distinct four-item subscales: horizontal collectivism (Cronbach α = 0.84) and vertical collectivism (Cronbach α = 0.80). A sample item is: “It is my duty to take care of my family, even when I have to sacrifice what I want.” To mitigate social desirability and common method biases prior to data collection, procedural remedies included ensuring participant anonymity, stating there were no right or wrong answers, separating predictor and outcome measures across distinct survey sections, and using pre-validated scales (Podsakoff et al., 2003).

### 3.3 Data analysis procedure

Statistical analyses were conducted in R (v4.3.2) using the lavaan (Rosseel, 2012) and semTools (Jorgensen et al., 2022) packages. To evaluate the psychometric properties of the measures, a two-stage analytical strategy was implemented. In line with established competency frameworks (Wiek et al., 2011), KCiS was conceptualized as an integrated, higher-order portfolio capability rather than a set of isolated skills. Operationalizing KCiS as a higher-order construct aligns with our theoretical focus on holistic competency integration, while maintaining structural model parsimony and preventing parameter inflation in complex moderated mediation analyses (Hair et al., 2021).

First, to empirically justify the higher-order structure, the hypothesized second-order CFA model (a higher-order KCiS construct with prosocial norms, vertical collectivism, horizontal collectivism and prosocial behavior) was compared against an unconstrained nine-factor correlated first-order model. Model fit across both specifications was evaluated against standard benchmarks as indicative reference guidelines: Comparative Fit Index [CFI: ≥0.90 acceptable, ≥0.95 good], Root Mean Square Error of Approximation [RMSEA: ≤ 0.08 acceptable, ≤ 0.05 good], and Standardized Root Mean Square Residual [SRMR: ≤ 0.08 acceptable, ≤ 0.05 good] (Byrne, 2016). When comparing measurement models, we used the change thresholds recommended by Chen (2007) for N > 200 [ΔCFI: ≤ 0.020 acceptable, ≤ 0.020 good; ΔRMSEA: ≤ 0.020 acceptable, ≤ 0.015 good, ΔSRMR ≤ 0.030 acceptable, ≤ 0.015 good], given that Δχ^2^ is oversensitive to sample size. These benchmarks were treated as indicative heuristics rather than absolute pass/fail rules, with model adequacy evaluated holistically in conjunction with parameter stability, factor loadings, and theoretical plausibility. Construct reliability and validity were established via Composite Reliability [CR ≥0.70], Average Variance Extracted (AVE ≥ 0.50), and Heterotrait-Monotrait ratios [HTMT: < 0.85 for conceptually completely distinct constructs; < 0.90 for highly related constructs or sub-dimensions] (Henseler et al., 2015). Additionally, factor score determinacy (FSD) coefficients were calculated to evaluate score determinacy for factor score imputation, with values exceeding 0.900 indicating that estimated factor scores highly accurately represent the underlying latent constructs (Grice, 2001).

Potential common method bias (CMB) was evaluated by introducing an unmeasured Common Latent Factor (CLF) to the baseline model with equalized paths (Podsakoff et al., 2003). Method bias was assessed by comparing model fit indices, total CLF variance against the 25.0% threshold (Williams et al., 1989), and unstandardized factor loading shifts against the 0.20 benchmark (Gaskin, 2012). Multi-group CFA was used to examine measurement invariance across the five universities, testing configural, metric, and scalar constraints for both first-order and second-order structures. Invariance was evaluated against the following thresholds recommended for N > 300: ΔCFI ≤ 0.010, ΔRMSEA ≤ 0.015, and ΔSRMR ≤ 0.030 for metric invariance, with SRMR ≤ 0.010 for scalar invariance (Chen, 2007). Achieving both metric and scalar invariance confirms that factor loadings and item intercepts operate equivalently across institutions, ruling out measurement bias and providing robust empirical justification for pooling the disaggregated individual-level data across all five universities without multi-group parameter estimation bias (Vandenberg and Lance, 2000).

Second, to avoid the severe model complexity, estimation bias, and convergence issues associated with estimating latent interactions approaches (e.g., Latent Moderated Structural equations [LMS] or product indicator approaches) when applied to complex higher-order constructs (Marsh et al., 2004), standardized latent factor scores were imputed from the validated measurement model. These factor scores were then used to estimate the first-stage moderated mediation model using 5,000 bias-corrected bootstrap samples to evaluate conditional indirect statistical associations.

Finally, to evaluate the stability of our findings against potential specification bias, we planned sensitivity analyses comparing our primary model against three alternative specifications: models with varying covariate controls (unadjusted vs. additional institutional covariates) and an alternative operationalization using unit-weighted mean composite scores

## 4 Results

### 4.1 Preliminary analysis

The baseline nine-factor first-order model demonstrated good fit [χ^2^(369) = 1358.39, *p* < 0.001; CFI = 0.929, RMSEA = 0.055, SRMR = 0.045]. The target second-order model exhibited virtually equivalent, acceptable fit [χ^2^(390) = 1613.14, *p* < 0.001; CFI = 0.913, RMSEA = 0.060, SRMR = 0.055]. No *post hoc* modifications were made, as no theoretically justified error covariances were identified from the modification indices.

**Figure 2:**
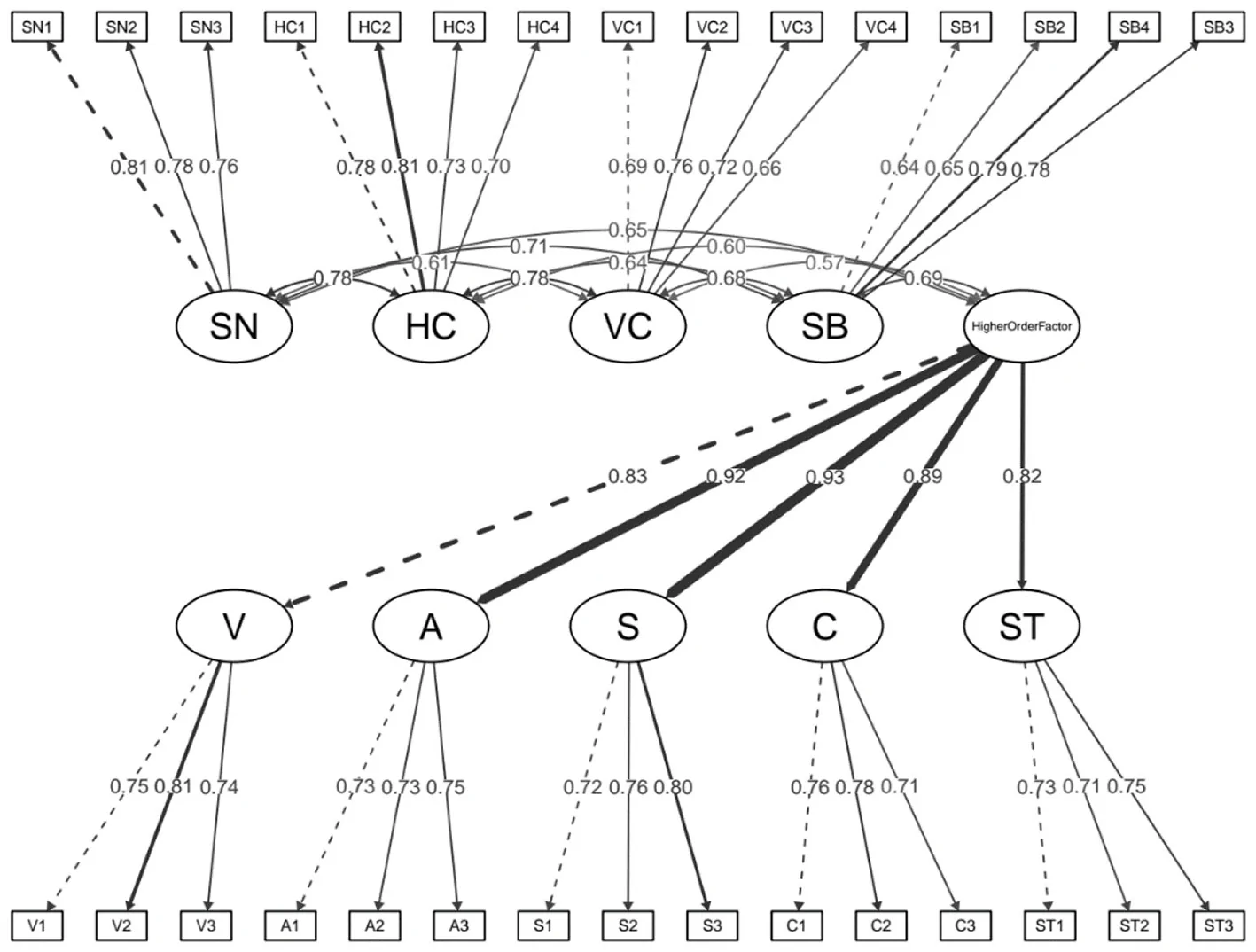
CFA diagram (second order).

Although imposing structural constraints on KCiS led to an expected shift in chi-square [Δχ^2^(21) = 254.75, *p* < 0.001], the change in relative fit statistics remained minor [ΔCFI = 0.016; ΔRMSEA = 0.005; ΔSRMR = 0.010], falling within established non-inferiority thresholds (Chen, 2007). This empirically supports the second-order specification of KCiS without compromising measurement quality. Figure 2 illustrates the CFA measurement model with standardized factor loadings (single-headed arrows) and inter-construct correlations (double-headed arrows). It shows that all loadings were strong (>0.60) and that the zero-order latent factor correlations were positive.

We then proceeded to assess convergent and discriminant validity. Table 1 presents descriptive statistics and convergent/discriminant validity measures of the focal constructs.

**Table 1:** Reliability and validity measures of focal constructs.

Construct	M (SD)	CR	AVE	HTMT Ratios									
				V	A	S	C	ST	PSN	HC	VC	PSB	KCiS
Values-Thinking (V)	3.65 (0.76)	0.81	0.59	1.00									
Anticipatory (A)	3.45 (0.75)	0.78	0.55	0.80	1.00								
Strategic (S)	3.43 (0.76)	0.80	0.57	0.73	0.87	1.00							
Collaboration (C)	3.34 (0.79)	0.79	0.56	0.69	0.78	0.85	1.00						
Systems-Thinking (ST)	3.69 (0.72)	0.77	0.53	0.72	0.70	0.73	0.75	1.00					
Prosocial Norms (PSN)	3.79 (0.69)	0.83	0.61	0.68	0.53	0.53	0.56	0.64	1.00				
Horizontal Collectivism (HC)	3.89 (0.67)	0.84	0.57	0.65	0.50	0.46	0.47	0.63	0.77	1.00			
Vertical Collectivism (VC)	3.82 (0.67)	0.80	0.51	0.52	0.62	0.48	0.52	0.59	0.62	0.81	1.00		
Prosocial Behavior (PSB)	3.45 (0.81)	0.81	0.52	0.56	0.61	0.67	0.70	0.59	0.70	0.59	0.68	1.00	
KCiS		0.94	0.77	-	-	-	-	-	0.65	0.60	0.59	0.68	1.00
Factor Score Determinancy	-	-	-	0.93	0.93	0.94	0.93	0.91	0.93	0.94	0.92	0.93	0.95

N = 883; CR, Composite Reliability, AVE, Average Variance Extracted.

Convergent validity was supported, as all constructs exhibited CR values above 0.70 and AVEs above 0.50. Discriminant validity was also established, as the HTMT ratios for distinct constructs (< 0.85) and sub-dimensions of KCiS (< 0.90) satisfied the established benchmarks (Henseler et al., 2015). We calculated the factor score determinacy coefficients for all measurement constructs. Additionally, factor score determinacy coefficients were calculated for all measurement constructs. All determinacy values exceeded 0.90, confirming that the imputed factor scores highly accurately reflect the true latent factors (Grice, 2001).

Before proceeding with the imputation of factor scores, we tested for CMB and metric invariance. When assessing potential CMB, the addition of a CLF to the baseline model yielded statistical improvements in model fit [Satorra Bentler scaled Δχ^2^ = 400.04, Δ*df* = 29, *p* < 0.001; ΔCFI = 0.049, ΔRMSEA = 0.022, ΔSRMR = 0.026]. However, such changes are expected given the sensitivity of chi-square tests to large samples and complex parameterization (Podsakoff et al., 2003). Crucially, the CLF accounted for only 23.9% of item variance-below the 25.0% threshold for problematic variance (Williams et al., 1989). Additionally, comparisons of the unstandardized factor loadings between the baseline model and the CLF model revealed no parameter differences exceeding the 0.20 threshold (Gaskin, 2012). Consequently, common method variance is unlikely to severely distort the observed relationships, albeit cross-sectional self-report limitations remain, supporting the retention of the unadjusted baseline model.

Finally, we assessed measurement invariance across the five universities using a multi-group CFA. First, the unconstrained baseline model was established, demonstrating acceptable fit [Δχ^2^ (1950) = 4254.970, *p* < 0.001; CFI = 0.848, RMSEA = 0.080, SRMR = 0.070]. A CFI below the 0.900 threshold is a known limitation in large highly-complex second-order CFA models with numerous indicators, where incremental fit indices are heavily penalized (Kenny and McCoach, 2003). Because absolute (RMSEA) and residual (SRMR) fit indices satisfied recommended criteria (Byrne, 2016) and information criteria consistently improved across constrained models [Akaike Information Criterion (AIC) configural = 58,587 vs. AIC 2nd-scalar = 58,400], the configural model was retained as an acceptable baseline for multi-group invariance testing. Next, first-order metric invariance was supported [Δχ^2^ (100) = 120.357, *p* = 0.081], and constraining measurement weights yielded minimal changes in fit [ΔCFI = −0.002, ΔRMSEA = −0.002, ΔSRMR = 0.007]. Constraining first-order item intercepts confirmed 1^st^-order scalar invariance, yielding a non-significant chi-square difference test [Δχ^2^ (80) = 88.139, *p* = 0.250], and no degradation in fit [ΔCFI = 0.000, ΔRMSEA = −0.001, ΔSRMR = 0.001]. Subsequently, constraining second-order factor intercepts confirmed second-order scalar invariance [Δχ^2^ (40) = 38.881, *p* =0.521; ΔCFI = 0.000, ΔRMSEA = −0.001, ΔSRMR = 0.001], satisfying all threshold criteria and confirming full measurement invariance across groups (Chen, 2007).

### 4.2 SEM modeling

The first-stage cross-sectional moderated mediation models with mean-centered variables produced a good fit for both Model A which has horizontal collectivism as moderator [χ^2^ (7) = 26.361, *p* < 0.001; CFI = 0.994, RMSEA = 0.056, SRMR = 0.037] and Model B which includes vertical collectivism as moderator [χ^2^ (7) = 19.401, *p* < 0.001; CFI = 0.995, RMSEA = 0.045, SRMR = 0.033]. It is worth noting that the chi-square statistic is an exact test of perfect fit and tends to be inflated in large samples, making it more likely to reject a model even if it fits reasonably well (Byrne, 2016). To ensure that parameter estimates were not biased by multicollinearity, particularly due to the interaction terms, Variance Inflation Factors (VIFs) were evaluated and these ranged from 1.012 to 4.605 for Model A and from 1.005 to 2.697 for Model B. These VIFs are below the recommended maximum threshold of 5.0 (Kline, 2023) and so, multicollinearity does not pose a threat to the stability or validity of the regression estimates or bootstrapped conditional associations. Summaries of moderated mediation results are exhibited in Tables 2,3.

**Table 2:** Moderated mediation results (horizontal collectivism).

Direct Relationships	*B* (S.E.)	z-value	p-value	95% *CI*	β
KCiS → PSN	0.35 (0.03)	10.53	< 0.01	(0.29,0.42)	0.27
PSN → PSB	0.43 (0.05)	9.33	< 0.01	(0.34,0.52)	0.44
HC → PSN	0.70 (0.03)	25.49	< 0.01	(0.64,0.75)	0.68
HC^*^KCiS → PSN	−0.02 (0.02)	−1.19	0.24	(−0.05,0.01)	−0.02
KCiS → PSB	0.49 (0.04)	12.65	< 0.01	(0.41,0.57)	0.38
Work Experience → PSN	−0.01 (0.02)	−0.62	0.54	(−0.06,0.03)	−0.01
Work Experience → PSB	−0.03 (0.03)	−1.32	0.19	(−0.09,0.02)	−0.03
Residency → PSN	−0.04 (0.02)	−1.69	0.09	(−0.08,0.01)	−0.03
Residency → PSB	0.04 (0.03)	1.27	0.20	(−0.01,0.09)	0.02
**Moderated Indirect Relationships**					
Low level of HC^a^	0.16 (0.02)	7.40	< 0.01	(0.12,0.20)	0.12
Mean Level of HC^b^	0.15 (0.02)	7.73	< 0.01	(0.11,0.19)	0.12
High Level of HC^c^	0.15 (0.02)	7.70	< 0.01	(0.11,0.18)	0.11
**Index of Moderated Mediation**	−0.01 (0.01)	−1.18	−0.24	(−0.02,0.01)	−0.01

KCiS, key competencies in sustainability; PSN, prosocial norms; HC, horizontal collectivism; PSB, prosocial behavior; Conditional explained variance: ^*a*^ *R*^2^ =0.078, ^*b*^ R^2^ = 0.073, ^*c*^ R^2^ = 0.069; R^2^ (Prosocial norms) =0.784, R^2^ (prosocial behavior)= 0.717.

**Table 3:** Moderated mediation results (vertical collectivism).

Direct Relationships	*B* (S.E.)	z-value	p-value	95% *CI*	β
KCiS → PSN	0.59 (0.04)	13.87	< 0.01	(0.51,0.68)	0.45
PSN → PSB	0.34 (0.03)	10.94	< 0.01	(0.27,0.40)	0.35
VC → PSN	0.45 (0.04)	11.04	< 0.01	(0.37,0.53)	0.40
VC^*^KCiS → PSN	−0.10 (0.03)	−3.91	< 0.01	(−0.16, −0.05)	−0.08
KCiS → PSB	0.38 (0.04)	10.54	< 0.01	(0.31,0.45)	0.30
Work Experience → PSN	0.04 (0.03)	1.27	0.20	(−0.02,0.09)	0.03
Work Experience → PSB	−0.03 (0.03)	−1.56	0.12	(−0.08,0.01)	−0.03
Residency → PSN	−0.04 (0.03)	−1.26	0.20	(−0.10,0.02)	−0.03
Residency → PSB	0.03 (0.03)	1.36	0.17	(−0.01,0.08)	0.02
**Moderated Indirect Relationships**					
Low level of VC^a^	0.22 (0.03)	8.77	< 0.01	(0.17,0.27)	0.18
Mean level of VC^b^	0.20 (0.02)	8.91	< 0.01	(0.15,0.25)	0.16
High Level of VC^c^	0.18 (0.02)	5.56	< 0.01	(0.14,0.22)	0.13
**Index of Moderated Mediation**	−0.03 (0.01)	−3.70	< 0.01	(−0.06, −0.02)	−0.03

KCiS, key competencies in sustainability; PSN, prosocial norms; VC, vertical collectivism; PSB, prosocial behavior; Conditional explained variance: ^*a*^ R^2^ = 0.248, ^*b*^ R^2^ = 0.202, ^*c*^ R^2^ = 0.161; R^2^ (Prosocial norms) = 0.630, R^2^ (prosocial behavior) = 0.767.

Tables 2, 3 show that in both Models, after controlling for work experience and residency, KCiS were positively associated with prosocial norms, prosocial norms were positively associated with prosocial behavior, and prosocial norms mediated the association between KCiS and prosocial behavior. This provided support for hypotheses H1 to H3 respectively. It is worth noting that the direct association between KCiS and prosocial behavior remained significant.

**Figure 3:**
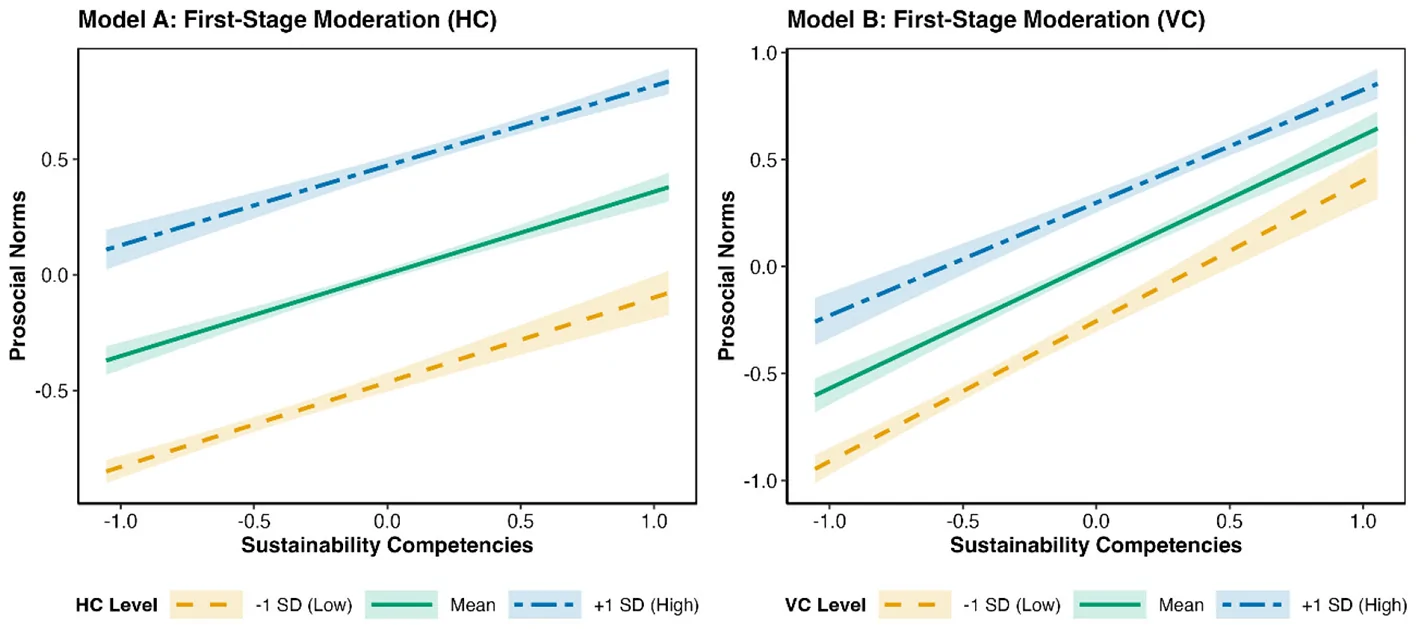
Interaction plots. Source: own study.

We then proceeded to examine first-stage moderation. Results showed that horizontal collectivism did not exhibit any significant moderation patterns, and so hypothesis H4a was rejected. However, vertical collectivism significantly moderated the association between KCiS and prosocial norms such that this association weakened at higher levels of vertical collectivism. Because H4b predicted an amplifying (positive) interaction, the statistically significant negative interaction means that hypothesis H4b was not supported. Instead, vertical collectivism exhibited an unanticipated dampening interaction, where higher vertical collectivism attenuated-rather than strengthened-the positive association between KCiS and prosocial norms. Figure 3 illustrates the simple slope interaction of KCiS and Collectivism (vertical and horizontal) on Prosocial Norms at low (-1SD) and high (+1SD) levels of the moderator, with 95% confidence bands.

Furthermore, the index of moderated mediation was not statistically significant for horizontal collectivism, thereby rejecting H5a. However, this index was statistically significant for vertical collectivism, indicating that vertical collectivism moderates the indirect association of KCiS with prosocial behavior via prosocial norms. Hypothesis H5b was not supported, since the indirect association diminished (rather than strengthened) as vertical collectivism increased.

### 4.3 Sensitivity analysis

To evaluate the robustness of our structural model, we conducted sensitivity analyses across four alternative specifications (Supplementary Table 2): Model 1 (primary model with two covariates and imputed factor scores), Model 2 (unit-weighted mean composite scores), Model 3 (three covariates including university), and Model 4 (unadjusted model without covariates). Across all four specifications, the direction and statistical significance of all direct, conditional indirect, and moderated mediation associations remained identical. While parameter estimates attenuated slightly under composite scoring (Model 2)-consistent with typical measurement error in non-latent scores-the inclusion or exclusion of control variables (Models 1, 3, and 4) yielded virtually identical estimates, confirming that our focal moderated mediation pattern is highly robust.

## 5 Discussion

### 5.1 Main findings

The present study examined how KCiS are related to prosocial behavior through prosocial norms and how this association is moderated by individual-level vertical collectivism. Consistent with hypotheses H1-H3, the results demonstrated that KCiS were positively associated with prosocial norms, which were positively associated with prosocial behavior, yielding a significant indirect association. These findings align with prior research indicating that individual competencies, knowledge, and awareness are correlated with higher prosocial attitudes, which co-occur with observable behavior (Bezzina et al., 2025; Morrison, 2011; Stern, 2000). In particular, KCiS appear to be positively linked with the cognitive and motivational foundations associated with prosocial norms consistent with Norm-Activation Theory (Schwartz, 1977), and with research highlighting the role of competencies in statistically corresponding to normative and behavioral responses (van der Linden et al., 2015; Wiek et al., 2011).

Contrary to expectations, vertical collectivism moderated the cross-sectional association between KCiS and prosocial norms in a negative direction; thus, Hypothesis H4b was rejected (not supported in its hypothesized positive direction). As a tentative *post-hoc* theoretical interpretation, this pattern may indicate a substitution or ceiling effect of collectivism (Gelfand et al., 2011). While prior theory suggested that collectivist settings characterized by structural hierarchy and compliance with authority would correspond to a stronger cross-sectional relationship (Triandis, 2001), our findings indicate a dampening interaction. Specifically, among individuals holding strong vertical collectivist values, one possible explanation is that prosocial norms co-occur with top-down social expectations, normative pressures, and shared group obligations, reducing the incremental statistical contribution of individual competencies. Additionally, this pattern may reflect a theoretical mismatch between in-group-focused vertical collectivism (prioritizing family or close peers) and wider, societal-level prosocial targets. In contrast, in lower vertical collectivism (more individualistic) settings, prosocial norms correspond more directly to internalized knowledge and personal capabilities, yielding a stronger association between individual competencies and norms (Hofstede et al., 2010). Importantly, this interaction represents a modest dampening interaction rather than a reversal of the association; KCiS remains positively associated with prosocial outcomes via prosocial norms across both low and high levels of vertical collectivism. Alternative explanations for this modest dampening interaction-such as slight ceiling effects (applying to an individual's personal value structure rather than macro-societal factors) in prosocial norm scores, in-group vs. generalized prosocial targeting, or subtle suppressor patterns-deserve further empirical scrutiny.

While this pattern aligns with cross-cultural research suggesting that behavior in collectivist settings is systematically aligned with external normative pressures (Oyserman et al., 2002), it should be noted that underlying psychological mechanisms such as normative conformity were not directly measured in this study. Further, the moderated mediation analysis confirmed that vertical collectivism attenuates the conditional indirect association between KCiS and prosocial behavior via prosocial norms, meaning Hypothesis H5b was not supported (as an amplifying interaction was predicted). Importantly, while vertical collectivism exhibited a modest dampening interaction, the indirect pathway from KCiS to prosocial behavior through prosocial norms remained positive and statistically significant across both low (-1SD) and high (+1SD) levels of vertical collectivism. This indicates that the competency-behavior association is culturally contingent. Among individuals with lower vertical collectivism, KCiS show a stronger positive association with prosocial norms and, correspondingly, behavior. In contrast, among individuals exhibiting higher vertical collectivism, the indirect association is weaker alongside a greater prevalence of internalized hierarchical social expectations and group-oriented motivations. These findings highlight the importance of incorporating individual cultural orientations, such as vertical collectivism, into models of sustainable behavior and extend prior research on how personal value systems are linked to the alignment between competencies and behavior (Oyserman et al., 2002; Schwartz, 2012).

Viewed holistically through Social Learning Theory, these patterns align with theoretical propositions regarding how observational learning, social modeling, and reinforcement processes are reflected in competency expression (Bandura, 1977, 1986). Rather than functioning in isolation, KCiS serve as learned cognitive structures through which students interpret social cues and evaluate prosocial actions. In highly vertically collectivist environments, however, strong normative expectations and visible modeling from authority figures or in-groups appear to diminish the marginal statistical association of individual-level competencies with norm expression. Consequently, while social learning processes correspond to the acquisition of both competencies and normative standards, the extent to which these competencies are aligned with personal norms remains culturally contingent.

### 5.2 Theoretical implications

This study extends NAT by conceptualizing sustainability competencies as distal statistical correlates associated with personal norm expression within a cross-sectional framework. While central activation mechanisms (awareness of consequences and ascription of responsibility) were omitted, the findings demonstrate that higher-order capabilities like KCiS are positively associated with the cognitive aspects of personal norm expression, reinforcing theoretically proposed process models sustainability behavior (van der Linden et al., 2015). The findings also refine theory on individual cultural orientation by showing that vertical collectivism negatively moderates-rather than positively amplifies-the cross-sectional statistical association between competencies and norms. This challenges assumptions that a collectivist orientation uniformly strengthens the alignment between personal competencies and socially-oriented attitudes. Instead, the results suggest that among individuals with a stronger vertical-collectivist orientation-characterized by hierarchy, duty, and compliance with group norms -, prosocial norms show a stronger positive alignment with externally imposed social expectations and group-oriented motivations than with individual capabilities (Oyserman et al., 2002; Schwartz, 2012). Crucially, this moderating interaction was specific to vertical collectivism, highlighting that structural compliance and deference to authority-rather than general peer-level togetherness (horizontal collectivism)-account for this dampening pattern.

Furthermore, Social Learning Theory serves as secondary interpretive perspective rather than a directly tested model. Although specific learning mechanisms (e.g., modeling, reinforcement) were not measured, Social Learning Theory provides a plausible framework for explaining how competencies and norms may develop in parallel (Bandura, 1977).

Finally, combining NAT with SLT perspectives responds to calls for multi-theoretical approaches, providing a more comprehensive account of how competencies, norms, and context co-occur alongside behavior (Iqbal and Piwowar-Sulej, 2023).

### 5.3 Practical implications

Because the design of this study is cross-sectional and observational, the following practical suggestions represent tentative hypotheses for instructional design and future intervention research rather than proven pedagogical strategies. Nevertheless, the pattern of cross-sectional associations offers important implications for higher education institutions and policymakers aiming to foster environments aligned with sustainability-oriented behavior among business and economics students.

First, our results suggest that higher self-reported KCiS is positively associated with prosocial behavior, an association that operates alongside the presence of personal prosocial norms. Educational programs should not only focus on knowledge and skills but also explicitly aim to foster conditions for prosocial norms, for example through reflective learning, ethical discussions, and real-world problem-solving activities (van Rijnsoever et al., 2023).

Second, experiential learning and active learning approaches represent promising contexts for further intervention research into these theoretical processes. Prior research shows that internships, service learning, and project-based activities correspond to students' ability to apply KCiS in practice (Aris et al., 2025; Meza Rios et al., 2018; Savage et al., 2015). Embedding such approaches in curricula may correspond to closer alignment between competencies and internalized norms.

Third, the findings highlight the importance of adopting culturally responsive and context-sensitive approaches to sustainability education. The observed dampening interaction of vertical collectivism suggests that in contexts characterized by strong social hierarchies or group compliance, personal competency development alone may show a more modest incremental association with individual norm expression. Higher education institutions operating across diverse cultural contexts should avoid one-size-fits-all curricula. Instead, instructional designers should test context-sensitive frameworks that leverage existing institutional structures, peer norms, and leadership models alongside individual competency building.

Overall, the study indicates that effective sustainability education is associated with aligning competencies, norms, and broader socio-cultural contexts, rather than focusing on competencies alone. Ultimately, designing sustainability curricula through a culturally responsive lens ensures that diverse educational environments can foster conditions where student competencies are strongly aligned with meaningful prosocial outcomes.

### 5.4 Limitations and future research directions

Despite its contributions, this study has several limitations that offer avenues for future research. First, the cross-sectional design precludes causal inferences, and the non-probability, self-selected sample of business and economics students - collected via an English-only survey across single institutions per country - limits generalizability beyond these specific cohorts. Additionally, data were geographically clustered within individual universities, and institutional clustering was not formally modeled via multi-level SEM.

Second, while using imputed CFA factor scores mitigates measurement error compared to raw summated scales, this two-step path approach does not fully partial out measurement error as simultaneous full SEM does. Furthermore, aggregating KCiS into a higher-order construct may mask variation and subtle associations among their underlying dimensions; hence further investigation is warranted (Bagozzi and Edwards, 1998).

Third, although NAT guided our conceptual framework, its core activation variables - Awareness of Consequences and Ascription of Responsibility - were not directly measured. Positioning KCiS as a framework factor offers a useful lens, but omitting these intermediate variables truncates the theoretical NAT pathway and prevents a direct test of the structural activation sequence.

Fourth, while our adapted prosocial norms scale demonstrated high internal consistency and strong item-total correlations, it incorporates both personal moral obligations and normative expectations of corporate responsibility. Although these dimensions naturally align among business and economics students, future research should consider empirically isolating individual-level moral obligations from institutional-level corporate expectations to test for potential sub-dimensional distinctions.

Finally, relying on self-reports introduces potential social desirability bias and common method variance, although procedural and statistical checks indicated minimal distortion. Future research should utilize longitudinal tracking, direct behavioral metrics, and explicit measures of specific social-learning processes (e.g., peer modeling and reinforcement) and norm-activation mechanisms (awareness of consequences and ascription of responsibility). Furthermore, future studies should explore whether alternative cultural dimensions (e.g., power distance) or boundary conditions (e.g., in-group vs. generalized prosocial targeting) further condition how vertical collectivism is associated with the alignment between competencies and norm activation.

## Data Availability

The raw data supporting the conclusions of this article will be made available by the authors, without undue reservation.

## References

[B1] Abyadh M. H. A. (2025). The influence of technostress and gratitude on university students' vitality: the roles of prosocial behaviours and psychological resilience. Int. J. Innov. Learn. 37, 149–178. doi: 10.1504/IJIL.2025.144197

[B2] Aris S. R. S. Wahi R. Zulkipli Z. A. Mohamed A. M. D. Hashim R. A. Elianawati. (2025). Factors influencing the sustainability of service learning (SULAM) projects: Insights from Malaysian university lecturers. Malays. J. Qual. Res. 11, 266–296. doi: 10.61211/mjqr110209

[B3] Bagozzi R. P. Edwards J. R. (1998). A general approach for representing constructs in organizational research. Organ. Res. Methods 1, 45–87. doi: 10.1177/109442819800100104

[B4] Bandura A. (1977). Social Learning Theory. Englewood Cliffs, NJ: Prentice-Hall.

[B5] Bandura A. (1986). Social Foundations of Thought and Action: A Social Cognitive Theory. Englewood Cliffs, NJ: Prentice-Hall.

[B6] Bandura A. (1991). “Social cognitive theory of moral thought and action,” in Handbook of Moral Behavior and Development, Vol. 1, eds W. Kurtines and J. L. Gewirtz (Hillsdale, NJ: Lawrence Erlbaum Associates), 45–103.

[B7] Bardi A. Schwartz S. H. (2003). Values and behavior: strength and structure of relations. Pers. Soc. Psychol. Bull. 29, 1207–1220. doi: 10.1177/0146167203254602 15189583

[B8] Barrio Campo S. Urionabarrenetxea S. San-Jose L. (2026). Embedding sustainability commitments in the strategic plans of European higher education institutions. Sustain. Dev. 34, e15525–e15540. doi: 10.1002/sd.71128

[B9] Barron P. Cord L. Cuesta J. Espinoza S. A. Larson G. Woolcock M. (2025). Social sustainability and the development process: what is it, why does it matter, and how can it be enhanced? Oxf. Dev. Stud. 53, 222–237. doi: 10.1080/13600818.2025.2502971

[B10] Bezzina F. Tracz-Krupa K. Zielińska A. Cassar V. (2025). Scale validation of the ‘Key Competences in Sustainability' indicator tool (KCiS-IT) among business and economics students in seven European HEIs. Int. J. Sustain. High. Educ. 26, 335–352. doi: 10.1108/IJSHE-09-2024-0621

[B11] Bryman A. (2016). Social Research Methods, 5^*th*^ *Edn*. London: Oxford University Press.

[B12] Byrne B. M. (2016). Structural Equation Modeling with AMOS: Basic Concepts, Applications, and Programming, 3rd Edn. New York, NY: Routledge. doi: 10.4324/9781315757421

[B13] Chang S. H. Wu P. Li H. Z. Jin X. Y. Zhong B. L. (2025). Temporal perspective and prosocial behavior under mortality salience: evidence from Chinese university students. Psychol. Res. Behav. Manag. 18, 1943–1953. doi: 10.2147/PRBM.S533218 40951368 PMC12433210

[B14] Chen F. F. (2007). Sensitivity of goodness of fit indexes to lack of measurement invariance. Struct. Equ. Modeling 14, 464–504. doi: 10.1080/10705510701301834

[B15] Cheng A. W. Rizkallah S. Narizhnaya M. (2020). “Individualism vs. collectivism,” *in The Wiley Encyclopedia of Personality and Individual Differences*, ed. B. J. Carducci (Hoboken, NJ: Wiley), 287–297. doi: 10.1002/9781118970843.ch313

[B16] Furlotti M. Vahidi G. Shipton H. (2022). Innovative behavior, emotional competencies, and experiential learning. Acad. Manage. Proc. 2022:143. doi: 10.5465/AMBPP.2022.143

[B17] Gaskin J. (2012). Common Method Bias. Gaskination's StatWiki. Available online at: http://statwiki.gaskination.com. (Accessed on August 4, 2026).

[B18] Gelfand M. J. Raver J. L. Nishii L. Leslie L. M. Lun J. Lim B. C. . (2011). Differences between tight and loose cultures: a 33-nation study. Science 332, 1100–1104. doi: 10.1126/science.1197754 21617077

[B19] Grice J. W. (2001). Computing and evaluating factor scores. Psychol. Methods 6, 430–450. doi: 10.1037/1082-989X.6.4.430 11778682

[B20] Hair J. F. Hult G. T. M. Ringle C. M. Sarstedt M. (2021). A Primer on Partial Least Squares Structural Equation Modeling (PLS-SEM), 3rd Edn. Thousand Oaks, CA: Sage Publications. doi: 10.1007/978-3-030-80519-7

[B21] Hayes A. F. (2017). Introduction to Mediation, Moderation, and Conditional Process Analysis: A Regression-Based Approach, 2nd Edn. New York, NY: Guilford Press.

[B22] Henseler J. Ringle C. M. Sarstedt M. (2015). A new criterion for assessing discriminant validity in variance-based structural equation modeling. J. Acad. Mark. Sci. 43, 115–135. doi: 10.1007/s11747-014-0403-8

[B23] Hidayah R. Solichah N. Chiedu C. Jannah M. Mu'awanah E. Bukhori B. . (2026). Exploring the influence of self-control, subjective wellbeing, happiness, and life satisfaction on prosocial behavior among Muslim students in Indonesia. Islamic Guid. Counc. J. 9:0020269727200. doi: 10.25217/0020269727200

[B24] Hoffman M. L. (2000). Empathy and Moral Development: Implications for Caring and Justice. Cambridge: Cambridge University Press. doi: 10.1017/CBO9780511805851

[B25] Hofstede G. (1984). Cultural dimensions in management and planning. Asia Pac. J. Manage. 1, 81–99. doi: 10.1007/BF01733682

[B26] Hofstede G. Hofstede G. J. Minkov M. (2010). Cultures and Organizations: Software of the Mind: Intercultural Cooperation and its Importance for Survival, 3rd Edn. New York, NY: McGraw-Hill.

[B27] Iqbal Q. Piwowar-Sulej K. (2023). Organizational citizenship behavior for the environment decoded: sustainable leaders, green organizational climate and person-organization fit. Balt. J. Manage. 18, 300–316. doi: 10.1108/BJM-09-2021-0347

[B28] Jorgensen T. D. Pornprasertmanit S. Schoemann A. M. Rosseel Y. (2022). semTools: Useful tools for structural equation modeling. R package version 0.5-6. Available online at: https://CRAN.R-project.org/package=semTools (Accessed June 4, 2026).

[B29] Kenny D. A. McCoach D. B. (2003). Effect of the number of variables on measures of fit in structural equation modeling. Struct. Equ. Modeling 10, 333–351. doi: 10.1207/S15328007SEM1003_1

[B30] Khalil O. Marouf L. Khalil N. (2021). Academics' knowledge sharing intentions and behaviours: the influence of espoused culture, social norm, and attitude. J. Inf. Knowl. Manage. 20:2150016. doi: 10.1142/S0219649221500167

[B31] Kim S. H. Kim S. (2022). The role of social norms on public service motivation and prosocial behavior: Moderating effect vs. direct effect. Int. J. Public Admin. 45, 1122–1131. doi: 10.1080/01900692.2021.1953072

[B32] Kline R. B. (2023). Principles and Practice of Structural Equation Modeling, 5th Edn. New York, NY: Guilford Press.

[B33] Markus H. R. Kitayama S. (1991). Culture and the self: implications for cognition, emotion, and motivation. Psychol. Rev. 98, 224–253. doi: 10.1037/0033-295X.98.2.224

[B34] Marsh H. W. Wen Z. Hau K. T. (2004). Structural equation models of latent interactions: evaluation of alternative estimation strategies and structural methodology. J. Educ. Behav. Stat. 29, 431–457. doi: 10.3102/10769986029004431 15355150

[B35] Meza Rios M. M. Herremans I. M. Wallace J. E. Althouse N. Lansdale D. Preusser M. (2018). Strengthening sustainability leadership competencies through university internships. Int. J. Sustain. High. Educ. 19, 739–755. doi: 10.1108/IJSHE-06-2017-0097

[B36] Morrison E. W. (2011). Employee voice behavior: integration and directions for future research. Acad. Manage. Ann. 5, 373–412. doi: 10.5465/19416520.2011.574506

[B37] Nikolskiy V. S. Ambarova P. A. Shabrova N. V. (2025). Prosocial attitudes and behavior of students: the effects of service learning. Vyssh. Obraz. Ross. 34, 9–30. doi: 10.31992/0869-3617-2025-34-3-9-30

[B38] Nilsson Dahlström Å. (2025). Challenges and opportunities for social sustainability in education: a scoping review. Cogent Soc. Sci. 11:2491706. doi: 10.1080/23311886.2025.2491706

[B39] Oyserman D. Coon H. M. Kemmelmeier M. (2002). Rethinking individualism and collectivism: evaluation of theoretical assumptions and meta-analyses. Psychol. Bull. 128, 3–72. doi: 10.1037/0033-2909.128.1.3 11843547

[B40] Piwowar-Sulej K. (2022). Sustainable development and national cultures: a quantitative and qualitative analysis of the research field. Environ. Dev. Sustain. 24, 13447–13475. doi: 10.1007/s10668-021-02011-w

[B41] Podsakoff P. M. MacKenzie S. B. Lee J. Y. Podsakoff N. P. (2003). Common method biases in behavioral research: a critical review of the literature and recommended remedies. J. Appl. Psychol. 88, 879–903. doi: 10.1037/0021-9010.88.5.879 14516251

[B42] Rey-Garcia M. Mato-Santiso V. (2020). Enhancing the effects of university education for sustainable development on social sustainability: the role of social capital and real-world learning. Int. J. Sustain. High. Educ. 21, 1451–1476. doi: 10.1108/IJSHE-02-2020-0063

[B43] Rosseel Y. (2012). lavaan: An R package for structural equation modeling. J. Stat. Softw. 48, 1–36. doi: 10.18637/jss.v048.i02

[B44] Savage E. Tapics T. Evarts J. Wilson J. Tirone S. (2015). Experiential learning for sustainability leadership in higher education. Int. J. Sustain. High. Educ. 16, 692–705. doi: 10.1108/IJSHE-10-2013-0132

[B45] Schoemann A. M. Boulton A. J. Short S. D. (2017). Determining power and sample size for simple and complex mediation models. Soc. Psychol. Personal. Sci. 8, 379–386. doi: 10.1177/1948550617715068

[B46] Schrage B. Maheshwari G. Velasquez S. (2025). Broadening the competencies of MBA students in Vietnam: integrating andragogical approaches with sustainable development goals. Int. J. Manag. Educ. 23:101217. doi: 10.1016/j.ijme.2025.101217

[B47] Schwartz S. H. (1977). “Normative influences on altruism,” in Advances in Experimental Social Psychology, Vol. 10, ed. L. Berkowitz (New York, NY: Academic Press), 221–279. doi: 10.1016/S0065-2601(08)60358-5

[B48] Schwartz S. H. (2012). An overview of the Schwartz theory of basic values. Online Read. Psychol. Cult. 2, 1–20. doi: 10.9707/2307-0919.1116

[B49] Siu A. M. H. Shek D. T. L. Law B. (2012). Prosocial norms as a positive youth development construct: a conceptual review. Sci. World J. 2012:832026. doi: 10.1100/2012/832026 PMC3361333 22666157

[B50] Stern P. C. (2000). Toward a coherent theory of environmentally significant behavior. J. Soc. Issues 56, 407–424. doi: 10.1111/0022-4537.00175

[B51] Triandis H. C. (2001). Individualism-collectivism and personality. J. Pers. 69, 907–924. doi: 10.1111/1467-6494.696169 11767823

[B52] Triandis H. C. Gelfand M. J. (1998). Converging measurement of horizontal and vertical individualism and collectivism. J. Pers. Soc. Psychol. 74, 118–128. doi: 10.1037/0022-3514.74.1.118

[B53] van der Linden S. Maibach E. Leiserowitz A. (2015). Improving public engagement with climate change. Perspect. Psychol. Sci. 10, 758–763. doi: 10.1177/1745691615598516 26581732

[B54] van Rijnsoever F. J. Sitzler S. Baggen Y. (2023). The change agent teaching model: Educating entrepreneurial leaders to help solve grand societal challenges. Int. J. Manag. Educ. 21:100893. doi: 10.1016/j.ijme.2023.100893

[B55] Vandenberg R. J. Lance C. E. (2000). A review and synthesis of the measurement invariance literature: suggestions, practices, and recommendations for organizational research. Organ. Res. Methods 3, 4–70. doi: 10.1177/109442810031002

[B56] Wang K. Ke Y. Sankaran S. (2024). The social pillar of sustainable development: measurement and current status of social sustainability of aged care projects in China. Sustain. Dev. 32, 227–243. doi: 10.1002/sd.2654

[B57] Wiek A. Withycombe L. Redman C. L. (2011). Key competencies in sustainability: a reference framework for academic program development. Sustain. Sci. 6, 203–218. doi: 10.1007/s11625-011-0132-6

[B58] Williams L. J. Cote J. A. Buckley M. R. (1989). Lack of method variance in self-reported affect and perceptions at work: reality or artifact? J. Appl. Psychol. 74, 462–468. doi: 10.1037/0021-9010.74.3.462

[B59] Woo E. Wooldridge M. LaPorte E. A. (2025). A case study on the effectiveness of cocurricular interdisciplinary sustainability programming for graduate students to create sustainability leaders. Int. J. Sustain. High. Educ. 26, 1–20. doi: 10.1108/IJSHE-06-2023-0239

[B60] Xu Z. Gao F. Fa A. Qu W. Zhang Z. (2024). Statistical power analysis and sample size planning for moderated mediation models. Behav. Res. Methods 56, 6130–6149. doi: 10.3758/s13428-024-02342-2 38308148

[B61] Zhou H. Ruan J. Xie J. Wang Y. Yang X. (2024). The effect of childhood maltreatment on the prosocial behavior of Chinese university students: a chain mediation analysis. Curr. Psychol. 43, 20722–20731. doi: 10.1007/s12144-024-05846-4

