# Supplementary material for: From competencies to action: unpacking the path from students' sustainability competencies to self-reported prosocial behavior
Source: Front Psychol. 2026 Sep 3;17:1918048. doi: 10.3389/fpsyg.2026.1918048 (PMC13582181; doi:10.3389/fpsyg.2026.1918048)
Supplement: Supplementary file 1 [file Supplementary_file_1.docx]

Table S1. Sensitivity Analysis of Horizontal Collectivism Model Across Alternative Covariate and Scoring Specifications

| **Path/Relationship** | **Model 1** | | **Model 2** | | **Model 3** | | **Model 4** | |
| --- | --- | --- | --- | --- | --- | --- | --- | --- |
| **Direct Relationships** | ***B* (S.E.)** | ***p-*value** | ***B (SE)*** | ***p-*value** | ***B (SE)*** | ***p-*value** | ***B (SE)*** | ***p-*value** |
| KCiS → PSN | .35 (.03) | < .01 | .32 (.04) | < .01 | .35 (.03) | < .01 | .35 (.03) | < .01 |
| PSN → PSB | .43 (.05) | < .01 | .29 (.04) | < .01 | .43 (.05) | < .01 | .42 (.05) | < .01 |
| HC → PSN | .70 (.03) | < .01 | .46 (.04) | < .01 | .70 (.03) | < .01 | .70 (.03) | < .01 |
| HC*KCiS → PSN | -.02 (.02) | .24 | -.03 (.02) | .09 | -.02 (.02) | .21 | -.02 (.02) | .23 |
| KCiS → PSB | .49 (.04) | < .01 | .37 (.04) | < .01 | .50 (.04) | < .01 | .49 (.04) | < .01 |
| **Conditional Indirect Relationships** |  |  |  |  |  |  |  |  |
| Low level of HC | .16 (.02) | < .01 | .10 (.02) | < .01 | .15 (.02) | < .01 | .15 (.02) | < .01 |
| Mean Level of HC | .15 (.02) | < .01 | .09 (.02) | < .01 | .15 (.02) | < .01 | .15 (.02) | < .01 |
| High Level of HC | .15 (.02) | < .01 | .09 (.02) | < .01 | .14 (.02) | < .01 | .14 (.02) | < .01 |
| **Index of Moderated Mediation** | -.01 (.01) | .24 | -.01 (.01) | .10 | -.01 (.01) | .22 | -.01 (.01) | .23 |

Note: KCiS = key competencies in sustainability, PSN = prosocial norms, HC = horizontal collectivism, PSB = prosocial behavior;

Model 1: Moderated mediation model with two covariates (work experience and residency) with imputed factor scores

Model 2: Moderated mediation model with two covariates (work experience and residency) with standardized unit-weighted mean composite scores

Model 3: Moderated mediation model with no covariates with imputed factor scores

Model 4 Moderated mediation model with three covariates (work experience, residency and university) with imputed factor scores.

Table S2. Sensitivity Analysis of Vertical Collectivism Model Across Alternative Covariate and Scoring Specifications

| **Path/Relationship** | **Model 1** | | **Model 2** | | **Model 3** | | **Model 4** | |
| --- | --- | --- | --- | --- | --- | --- | --- | --- |
| **Direct Relationships** | ***B* (S.E.)** | ***p-*value** | ***B (SE)*** | ***p-*value** | ***B (SE)*** | ***p-*value** | ***B (SE)*** | ***p-*value** |
| KCiS → PSN | .59 (.04) | <.01 | .43 (.04) | < .01 | .59 (.04) | < .01 | .59 (.04) | < .01 |
| PSN → PSB | .34 (.03) | <.01 | .26 (.04) | < .01 | .33 (.03) | < .01 | .33 (.03) | < .01 |
| VC → PSN | .45 (.04) | <.01 | .26 (.04) | < .01 | .50 (.04) | < .01 | .45 (.04) | < .01 |
| VC*KCiS → PSN | -.10 (.03) | <.01 | -.06 (.02) | < .01 | -.10 (.03) | < .01 | -.10 (.03) | < .01 |
| KCiS → PSB | .38 (.04) | <.01 | .32 (.04) | < .01 | .39 (.04) | <.01 | .37 (.04) | < .01 |
| **Conditional Indirect Relationships** |  |  |  |  |  |  |  |  |
| Low level of VC | .22 (.03) | <.01 | .13 (.02) | < .01 | .22 (.03) | < .01 | .22 (.03) | < .01 |
| Mean Level of VC | .20 (.02) | <.01 | .11 (.02) | < .01 | .20 (.02) | < .01 | .20 (.02) | < .01 |
| High Level of VC | .18 (.02) | <.01 | .10 (.02) | < .01 | .18 (.02) | < .01 | .18 (.02) | < .01 |
| **Index of Moderated Mediation** | -.03 (.01) | <.01 | -.02 (.01) | < .01 | -.04 (.01) | < .01 | -.03 (.01) | < .01 |

Note: KCiS = key competencies in sustainability, PSN = prosocial norms, VC = vertical collectivism, PSB = prosocial behavior;

Model 1: Moderated mediation model with two covariates (work experience and residency) with imputed factor scores

Model 2: Moderated mediation model with two covariates (work experience and residency) with standardized unit-weighted mean composite scores

Model 3: Moderated mediation model with no covariates with imputed factor scores

Model 4: Moderated mediation model with three covariates (work experience, residency and university) with imputed factor scores.
